# Supplementary material for: Screening, Identification, and Fermentation Condition Optimization of a High-Yield 3-Methylthiopropanol Yeast and Its Aroma-Producing Characteristics
Source: Foods. 2024 Jan 27;13(3):418. doi: 10.3390/foods13030418 (PMC10855053; doi:10.3390/foods13030418)
Supplement: Supplementary file 1 [file foods-13-00418-s001.zip › foods-2805565-supplementary.pdf]

Supplementary material for

Identification and fermentation condition optimization of a high-yield 3-methylthiopropanol yeast

Table S1. Factors and levels of single factor design and their optimization conditions.

Factor	Level/type	Optimization condition
Glucose concentration (g/L)	10, 15, 20, 25, 30, 35, 40, 45, 50, 55 and 60	**40**
Yeast extract concentration (g/L)	0, 0.4, 0.8, 1.2, 1.6 and 2.0	**0.4**
Surfactant type	Blank, glycerol, Tween 20, Tween 40, Tween 60, Tween 80, and Triton 100	**Tween 80**
Tween 80 concentration (g/L)	0, 2, 4, 8, 16, 32 and 64	**2**
L-methionine concentration (g/L)	0, 2, 4, 6, 8 and 10	**4**
L-methionine addition time (h)	0, 12, 24, 36, 48, 60 and 72	**0**
Temperature (°C)	20, 24, 28, 32, 36 and 40	**32**
Initial pH	3, 3.5, 4, 4.5, 5, 5.5, 6, 6.5 and 7	**5**
Inoculum size (%)	0.1, 0.2, 0.4, 0.8, 1.6, 3.2 and 6.4	**0.8**
Shaking speed (rpm)	0, 45, 90, 135, 180, 225 and 270	**225**
Liquid volume (mL)	25, 50, 75, 100 and 125	**25**
Fermentation time (h)	0, 12, 24, 36, 48, 60, 72, 84 and 96	**60**

Table S2. Physiological and biochemical characteristics of strain Y1402.

Test	Results		Test	Results	
	**Sugars**	**Characteristics**		**Carbon sources**	**Characteristics**
Sugar fermentation	L-Rhamnose	No acid, no gas, no growth	Carbon source assimilation	Ethanol	Good growth
	D-Galactose	Acid production, no gas, good growth		Glycerol	Growth
	D-Maltose	Acid production, no gas, good growth		D-Trehalose	No growth
	D-Xylose	No acid, no gas, no growth		D-Raffinose	No growth
	D-Arabinose	No acid, no gas, no growth		Inulin	Growth
	Glucose	Acid and gas production, good growth		Mannitol	No growth
	Lactose	No acid, no gas, no growth		D-Ribose	Good growth
	Sucrose	Acid production, no gas, good growth		D-Sorbitol	No growth
	**Nitrogen sources**	**Characteristics**		**Nitrogen sources**	**Characteristics**
Nitrogen source assimilation	Urea	Good growth	Nitrogen source assimilation	Potassium nitrate	Growth
	Ammonium sulfate	Good growth		L-Phenylalanine	Good growth

Sodium nitrite	Growth	L-Lysine	Good growth
Hydrogen sulfide test	Negative	Indole test	Positive
Citrate test	Positive	Methyl red test	Positive
Urea test	Negative	Voges-Proskauer test	Negative
Starch hydrolysis test	Positive	Gelatin liquefaction test	Negative

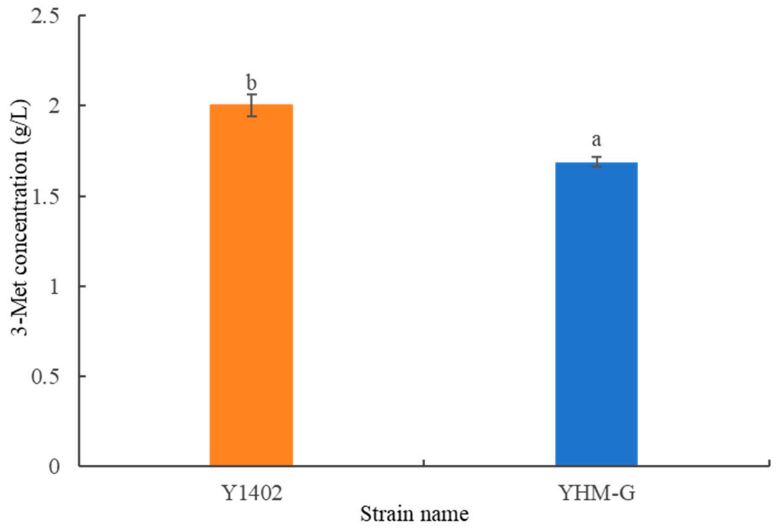

Figure S1. Comparison of 3-Met produced by yeast Y1402 and YHM-G.

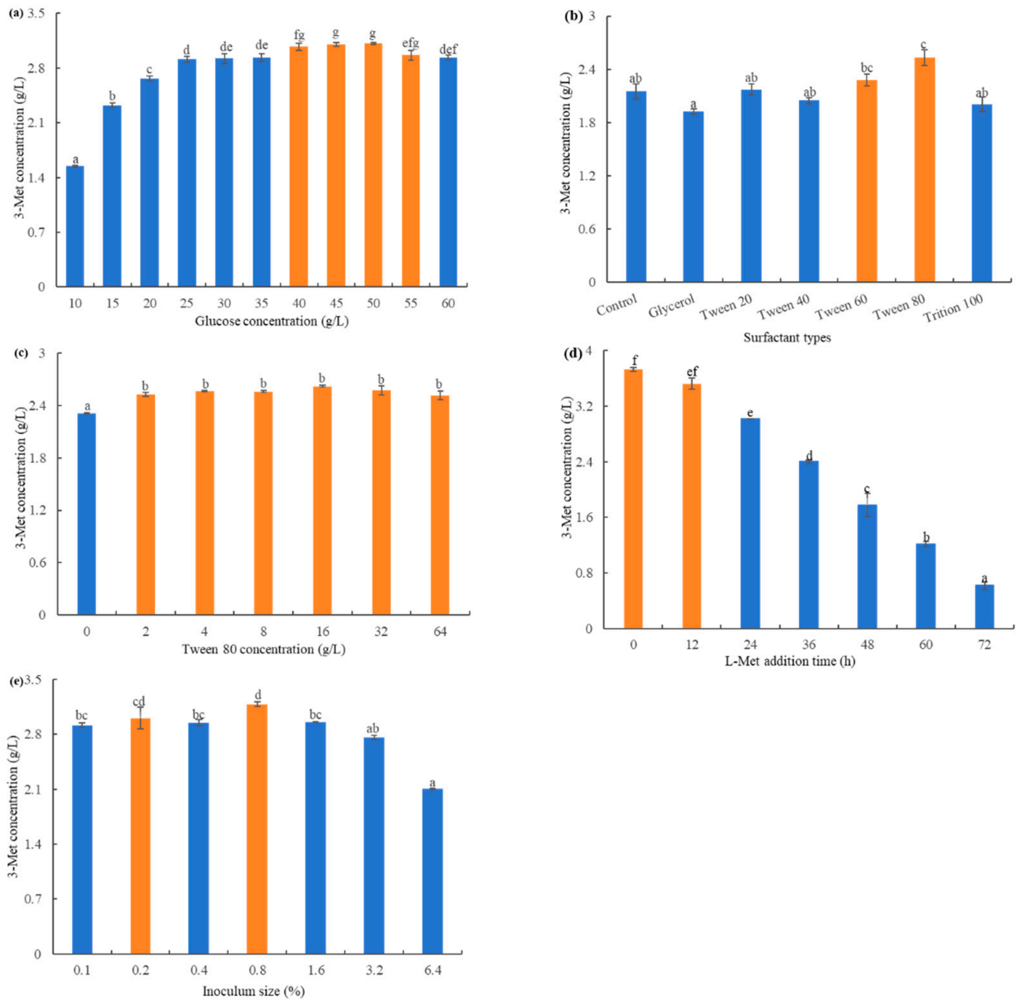

Figure S2. Effect of glucose concentration (10, 15, 20, 25, 30, 35, 40, 45, 50, 55 and 60 g/L) (a), surfactant types (control, glycerol, Tween 20, Tween 40, Tween 60, Tween 80 and Triton X-100) (**b**), Tween 80 concentration (0, 2, 4, 8, 16, 32 and 64 g/L) (**c**), L-Met addition time (0, 12, 24, 36, 48, 60 and 72 h) (**d**), inoculum size (0.1, 0.2, 0.4, 0.8, 1.6, 3.2 and 6.4%, v/v) (**e**) on 3-Met concentration by Y1402. Same letters in the column indicates that the data do not differ significantly at 5% probability by the Tukey test. The orange bar means the best conditions on 3-Met concentration.

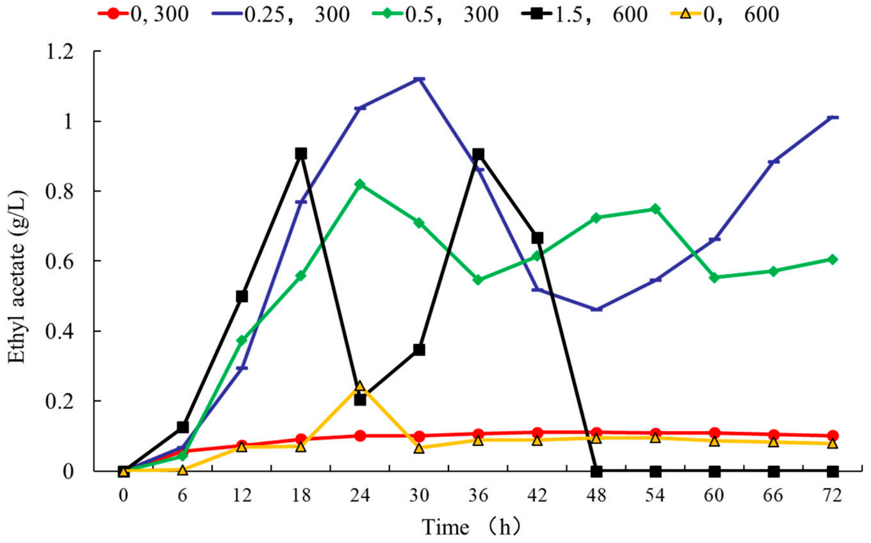

Figure S3. The yield of ethyl acetate at different conditions at *S. cerevisiae* Y3401 and *W. anomalus* Y3604 ratio of 1:1 by simultaneous mixed fermentation in a 5 L fermenter. Note: 0, 300: the aeration rate of the fermenter is 0 vvm and the agitation speed is 300 rpm; 0.25, 300: the aeration rate of the fermenter is 0.25 vvm and the agitation speed is 300 rpm; 0.5, 300: the aeration rate of the fermenter is 0.25 vvm and the agitation speed is 300 rpm; 1.5, 600: the aeration rate of the fermenter is 1.5 vvm and the agitation speed is 600 rpm; 0, 600: the aeration rate of the fermenter is 0 vvm and the agitation speed is 600 rpm.
